# Dual-Task-Based Music Therapy to Improve Executive Functioning of Elderly Patients with Early Stage Alzheimer’s Disease: A Multiple Case Study

**DOI:** 10.3390/ijerph191911940

**Published:** 2022-09-21

**Authors:** Soo Ji Kim, Jin-Kyoung Park, Myung Sun Yeo

**Affiliations:** 1 Music Therapy Education, Graduate School of Education, Ewha Womans University, Seoul 03760, Korea; 2 Arts Therapy Education Institute, Ewha Womans University, Seoul 03760, Korea; 3 Department of Music Therapy, Graduate School, Ewha Womans University, Seoul 03760, Korea

**Keywords:** dual-task, music therapy, early stage Alzheimer’s disease, executive functioning, drum playing

## Abstract

Deficits in executive functioning are a common feature of Alzheimer’s disease (AD) and result from impairment in the central executive system. As a result, elderly patients with early stage AD may require interventions that are more cognitively intense than traditional interventions. To address this need, in this multiple case study, we explored a dual-task-based music therapy intervention that involved drum playing and singing designed to induce attentional and motor controls. Three octogenarians diagnosed with early stage AD participated in 12 dual-task-based music therapy sessions over 6 weeks. Measures of executive functioning and the performance of a bimanual drum tapping task were evaluated before and after the intervention. Improvements in executive functioning were observed for participants A and C. After the intervention, reduced mean synchronization errors were found for the simultaneous tapping condition for all three participants. Although there was variability in the functional changes between participants, it is noteworthy that positive improvements in the elderly patients with early stage AD were obtained following dual-task-based music therapy. The results suggest that music therapy integrated into the dual-task paradigm can be an effective way to address degenerative cognitive deficits among elderly patients with early stage AD.

## 1. Introduction

People worldwide are living longer, and disorders associated with aging are on the rise. For example, the latest estimate of the number of people affected by dementia worldwide is 55 million [1], and the percentage of people with dementia is now over 10% in Korea [2]. Considering the detrimental health and financial impacts of dementia [1], it has become a global priority to seek ways to ameliorate symptoms. Individuals with dementia experience the progressive loss of cognitive, physical, and functional ability beyond that accounted for by normal aging processes [3]. Accordingly, there is an urgent need for the early diagnosis and intervention in dementia [4].

In Korea, the most prevalent subtype of dementia is Alzheimer’s disease (AD) [5], and cognitive decline is the most common characteristic of AD [6]. In particular, impairment in executive functioning (EF) is an early marker of AD [7,8]. EF is the set of skills that allows a human being to perform specific cognitive actions, including processing and evaluating, planning, and executing tasks; self-monitoring; and self-regulating [9], and individuals with AD have a deterioration in EF, affecting all of these processes [10]. Because EF involves higher-order functions that allow for behavior modification and adjustment in response to environmental and contextual changes [11], inhibitory control is critical to perform cognitive tasks. Early stage AD is characterized by the onset of specific symptoms and challenges in independent living, including difficulty maintaining proper personal hygiene and interpersonal relationships. In addition, deterioration in EF occurs more rapidly than the loss of other cognitive functions in individuals with AD [12,13]. 

The most prominent cognitive changes in early stage AD are impairments in attentional and inhibitory control [14], which can be enhanced by dual-task processing, including task switching [15]. Compared to single-task performance, dual-task performance challenges attentional capacities that simultaneously use two different functional areas: motor and cognitive [16,17,18,19]. Due to degeneration in the central nervous system, patients with AD have difficulty performing dual tasks [20].

A dual-task paradigm incorporating a concurrent task with rhythmic cueing has been introduced as an effective intervention strategy in the cognitive rehabilitation process for older adults [21,22,23]. Bimanual instrument playing can be a means of implementing the dual-task paradigm because auditory–motor interaction occurs during upper limb movement to make sound through an instrument. Previous studies showed that functional coupling between auditory and motor areas of the brain results in cognitive activation for movement planning and execution during musical instrument playing [24,25,26]. In particular, bimanual instrument playing with rhythmic cues with tempo alterations involves executive control and cognitive flexibility for older adults [22].

Much research supports the positive effects of music interventions on cognitive and motor functions [27,28,29]. Among the various types of music interventions, instrument playing can be tailored to target specific cognitive domains [21,22], and the complexity of the task can be altered using various motor experiences during the music-making process. The increased attentional demands associated with using external rhythmic cues can be a therapeutic strategy for further increasing the cognitive intensity of the dual-task intervention for individuals with AD, who often experience difficulty with motor planning and have problems with attentional capacity [20]. Dual-task attributes in previous studies involved gait or tapping as the motor functions and simple numeric or verbal tasks for the cognitive functions to manipulate cognitive loads. Drum playing, a bimanual motor movement, coupled with auditory feedback may activate cognitive functioning. In a dual-task situation, bimanual coordination can be varied with task complexity (i.e., level of involvement in the drum playing task) [30].

In this study, to observe changes in the EF of elderly patients (over the age of 80 years) with early stage AD, music therapy was formulated based on the dual-task paradigm. Rhythmic cueing was utilized to change the bimanual limb movements and tempo during drum playing. To enhance intrinsic motivation and engagement levels, live musical accompaniment and singing were employed in the intervention. In previous studies, dual-task-based drum playing was found to be an efficient therapeutic strategy combining upper extremity motor control with attentional control using musical components, such as rhythm pattern or tempo [31]. A recent study of a drum-playing intervention based on the dual-task paradigm for individuals with Parkinson’s disease found improvements in attentional control and motor functions in the participants’ dual-task performance [31].

Considering the impact of the dual-task strategy on the cognitive functioning of cognitively impaired older adults [32], more efficient means for facilitating dual-task performance with this population are urgently needed. As such, this multiple case study investigated the effect of a dual-task-based music therapy intervention on the cognitive functioning of elderly patients with early stage AD.

## 2. Materials and Methods

### 2.1. Participants

The participants in this study were recruited between July 2019 and June 2021 from a residential facility for patients with dementia located in Gyeonggi-do, South Korea. Three patients (M = 2, F = 1) over the age of 80 years old and with early stage AD participated in this study. The participants’ demographic information is displayed in Table 1.

The inclusion criteria used in this study were the following: at least 60 years of age, a diagnosis from a neurologist of early stage AD, no diagnosis of brain damage or any psychiatric disorder other than dementia, and a score below 18 on the Geriatric Depression Scale (GDS) [33].

### 2.2. Procedure

Each participant engaged in 40 min individual sessions twice a week, for a total of 12 sessions over 6 weeks. The intervention was conducted in a quiet place at the participant’s residential facility to comply with COVID-19 protocols at the time of data collection. We provided the participants and their primary caregivers with information regarding the program content, the study duration, the procedures to be used, the expected benefits and risks of participation in the study, and confidentiality. Each participant provided written consent prior to their participation. During the intervention, local COVID-19 protocols required the participants and researchers to wear a certified KF-94 face mask. At the start of each session, the parties disinfected their hands, and the participant’s temperature was checked. Additionally, the room was disinfected after each session. In addition, the researchers participated in COVID-19 training before participating in the study and took weekly PCR tests during the research period. The Institutional Review Board at Ewha Woman’s University approved all procedures and ethical issues related to this study (IRB No. 201909-0025-05).

### 2.3. Measurements of Cognitive Functioning

The contrasting and Go/No-Go tests were used to assess EF [34]. The Trail Making Test (TMT) [35] was also used to measure EF as well as working memory, processing speed, visual attention, and cognitive flexibility [36]. The TMT is divided into two tasks: TMT-A and TMT-B. Part A instructs participants to connect 15 numbered circles in sequential order, and Part B instructs them to connect 15 circles alternating between sequential numbers and words (e.g., 1—Monday, 2—Tuesday, and so on). Both Parts A and B measure cognitive flexibility. However, TMT-B also requires attentional control, inhibition function, and set-shifting task performance [37]. The Fist–Edge–Palm task and the alternating hand movement task were used to assess frontal dysfunction [38]. For the Fist–Edge–Palm task, participants were asked to successively follow hand postures: (a) a vertically placed fist (fist), (b) a vertically placed palm (edge), and (c) a horizontally placed palm (palm) [39]. The alternating hand movement task assessed the execution of repeatedly clenching one hand while unfolding the other at the same time and then reversing hands [39].

In addition, the Korean version of the Cognitive Impairment Screening Test (K-CIST) was administered. This is a cognitive measurement developed by the Korean Ministry of Health and Welfare that asks questions reflecting cultural sensitivity. The K-CIST consists of 13 questions that are divided into the following domains: orientation, attention, visuospatial function, executive function, memory, and language function. The total score is calculated by adding the scores for each question; the lowest possible score is 0, and the highest possible score is 30 [40].

The Subjective Memory Complaints Questionnaire (SMCQ) a self-reporting questionnaire that asks about general memory problems, was also administered. On the questionnaire, 4 of the 14 items focus on the individual’s subjective judgment of memory impairment, and the other 10 items assess everyday memory [41]. Finally, the GDS was administered to evaluate the degree of depressive mood in potential participants [33]. The GDS contains 30 questions. A score of 18 or above indicates depression [42].

### 2.4. Measurements of the Bimanual Drum Tapping Task

In this study, the musical instrument digital interface (MIDI) drum tapping task was used. The drum tapping measurement consisted of two tasks: simultaneous tapping and alternative tapping [21]. In the first task, each participant was instructed to tap a drum with both hands simultaneously at their preferred tempo. In the second task, participants tapped the drum in time to the regular rhythmic cueing provided. The tasks involved at least 20 taps for each trial. In addition, the tempo of the rhythmic cueing was adjusted according to five conditions: self-paced tempo (baseline) and adjusted tempo at ±10 and ±20% of the baseline tempo for each participant. Additionally, participants were instructed to maintain the task until they were told to stop.

**Figure 1:**
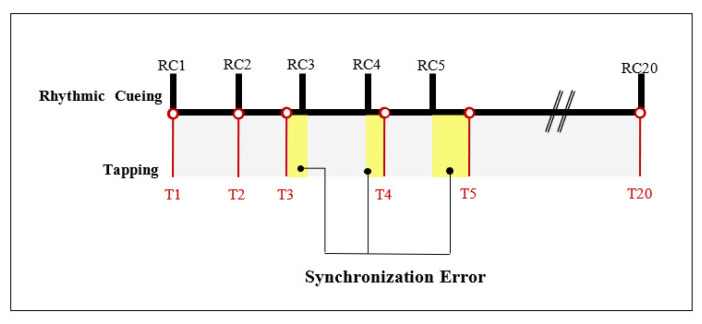
The measurement paradigm of the MIDI drum tapping task. The synchronization error was calculated as the time between rhythmic cueing and drum tapping.

The drum tapping task device was a 12-inch electronic drum (Alesis, Cumberland, RI, USA). The MIDI AMON (Infrasonic, Incheon, Korea) was used to transfer the signals obtained during drum tapping to a Cubase 8.5 (Steinberg Media Technologies AG, Hamburg, Germany) assessment device. The signals of the MIDI electronic drum allowed for the collection and analysis of the timing accuracy data related to the synchronization error between metric rhythmic cueing and tapping (see Figure 1). In addition, during the provision of rhythmic cueing, the metronome in the Cubase 8.5 software was used.

### 2.5. Dual-Task-Based Music Therapy

The music therapy intervention consisted of drum playing with rhythmic cueing based on the dual-task paradigm. As such, the dual-task-based music therapy intervention included a timing cue for movement to promote cognitive flexibility during the participants’ drum playing with different types of bimanual movements. The tempo for cueing the bimanual movements changed by ±10 and ±20 modulation of each participant’s self-paced tempo to facilitate attentional and inhibitory controls during drum playing. The dual-task-based music therapy intervention consisted of three stages (see Table 2). In Stage 1, participants were asked to engage in respiratory and muscle relaxation movements. In Stage 2, the participants played their drums based on the dual-tasks. The tasks of this second stage consisted of two activities: (a) drum playing with regular rhythmic cueing and (b) phased drum-playing tasks according to varied rhythmic cueing. Finally, in Stage 3, tasks involved chanting with rhythmic cueing and therapeutic singing.

## 3. Results

### 3.1. Changes in Cognitive Functioning for Each Participant

The outcomes of the EF assessments (i.e., contrasting task, Go/No-Go task, Fist–Edge–Palm task, alternating hand movement task, TMT-A test, and TMT-B test) are presented in Table 3. First, the results show that participant A improved the most in terms of EF compared to their baseline performance. For participant B, the results showed improvement in her contrasting test score and executive time on the TMT-A. Her executive time on the TMT-A decreased from 214.0 s to 104.37. However, participant B’s score for the TMT-B was invalid due to her execution time being over 300 s, which is the maximum time before an individual is deemed to have failed the test. Similarly, participant B failed the Fist–Edge–Palm and alternating tasks. Finally, participant C had a perfect score of 20 on the contrasting task at both baseline and in the post-test. Participant C’s performance on the Go/No-Go test increased from 9 to 11. In addition, their executive time on the TMT-A decreased from 35.62 to 27.27. In contrast, the TMT-B for participant C increased by 49.43 s.

### 3.2. Changes in Bimanual Drum Tapping with Rhythmic Cueing

The bimanual drum tapping task with rhythmic cueing was conducted to examine attentional control as a compensatory strategy to maintain interlimb coordination, and matching to rhythmic cueing involved a response to external stimuli. The stability of motor tasks is found during synchronized bimanual tapping with rhythmic cueing. The mean synchronization errors during bimanual tapping across the five conditions (self-paced, +10% tempo, +20% tempo, −10% tempo, and −20% tempo) were measured at pre-test (baseline) and post-intervention.

**Figure 2:**
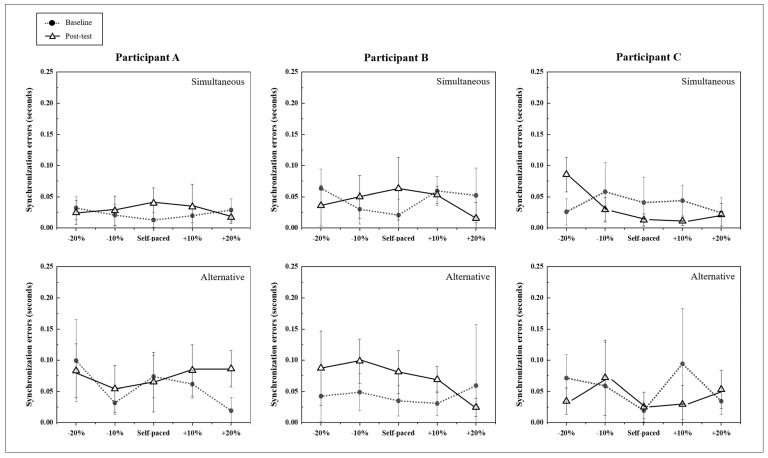
Changes in synchronization errors for the bimanual drum tapping task. The mean synchronization errors during bimanual tapping across the five conditions (self-paced, +10% tempo, +20% tempo, −10% tempo, and −20% tempo) were measured pre- and post-intervention for each participant.

The degree of synchronization errors for all three participants showed inconsistent changes in each tempo condition (see Figure 2 and Table 4); however, the overall tendency was for synchronization errors to increase more for the alternative tapping condition than for the simultaneous tapping condition in the post-test.

It is important to note that participant A, who demonstrated improved test scores on all EF measurements after the intervention, maintained small synchronization errors for all tempo conditions with simultaneous tapping tasks after the intervention, but variability was found in this participant’s synchronization errors for the alternative tapping condition.

Participant B, who failed several EF tests, had decreased synchronization errors for the −20 tempo and +20 tempo simultaneous tapping conditions after the intervention. Additionally, for the +20% tempo condition of the alternative tapping task, participant B’s synchronization error decreased by 0.029 s.

Participant C, who was the oldest participant, had synchronization errors that decreased during the simultaneous tapping tasks, and for four conditions (−10%, self-paced, +10%, and +20%), this participant showed smaller synchronization errors after the intervention. However, participant C’s synchronization errors varied the most during alternative tapping.

### 3.3. The Ratio of Each Type of Drumming within the Intervention

**Figure 3:**
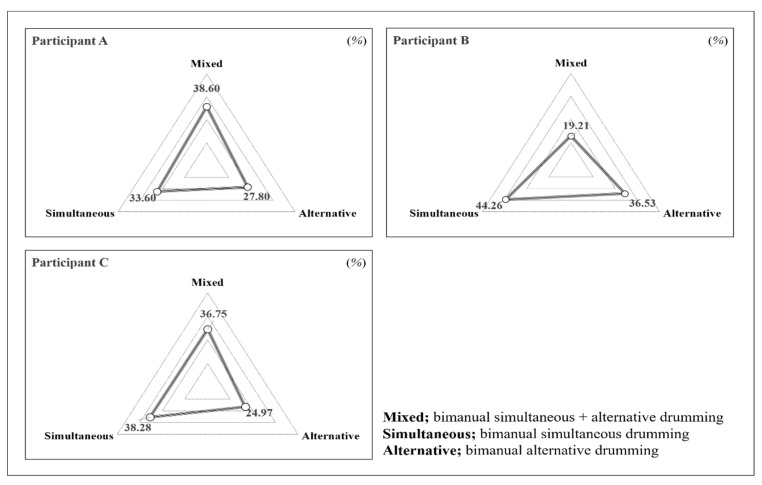
The ratio of each type of drum playing (i.e., simultaneous, alternative, or mixed) across the 12 sessions of the intervention.

In this study, we analyzed the ratio of each type of bimanual drum playing for each participant to identify individual differences at the level of engagement. The types of bimanual drum playing were categorized into simultaneous drumming, alternative drumming, or mixed types of drumming. We observed video recordings of each session of each participant and measured the time each participant spent engaged in each type of drum playing (i.e., simultaneous, alternative, or mixed playing) in each session. We then calculated the total time spent during the intervention on each of the three types of bimanual drum playing and divided this sum by the total session time to obtain the average ratio across the 12 sessions for each participant. The ratio of each type of bimanual drumming is displayed in Figure 3.

First, participant A showed the highest mixed drumming rate (38.6%) and the lowest execution rate of simultaneous drumming (33.6%). Participant B showed the highest rates of simultaneous drumming (44.26%) and alternative drumming (36.53%). Conversely, participant B’s execution rate of mixed drumming was the lowest (19.21%) among the participants. Participant C showed comparable simultaneous drumming (38.28%) and mixed drumming (36.75%) rates but had the lowest alternative drumming rate (24.97%).

## 4. Discussion

This case study found changes in cognitive and motor performance following a dual-task-based music therapy intervention for elderly patients with early stage AD and identified the individual differences observed based on the participants’ level of intervention engagement. It is clinically meaningful to address changes in EF that may be related to the time participants spent engaged in the specific types of drum playing and changes in cognitive measures. In addition, this study included octogenarians, who are generally not included in clinical studies.

This multiple case study is meaningful because it presents outcomes for octogenarians, who are a growing population, and it is the first study to employ bimanual drum playing and the dual-task paradigm with this age group. The three cases in this study suggest ways in which dual-task-based music therapy can be applied in AD interventions, especially for elderly patients. The results of this study also show that constant attention to rhythmic cueing can eventually lead to increased timing accuracy in the auditory system’s automatic representation of sound, which leads to enhanced perceptions of movement timing [43].

All three of the participants involved in the 6-week dual-task-based music therapy intervention focusing on frontal cognitive stimulation and flexibility performed better on the contrasting task and TMT-A test. In particular, participant A showed improvements on all cognitive measures, while participant B seemed to have challenges with the Go/No-Go and TMT-B. Despite the inconsistency in the changes across the cognitive measures, the influence of the intervention can be addressed. Music tasks in this study included drum tapping with demands of varying complexity for attentional processes, and this type of drum tapping may provide a form of cognitive demand and stimulus to drive cognitive network processes in the brain to improve cognitive flexibility for individuals with early stage AD. Timing cues in music provide movement anticipation and an opportunity to use attentional control. This is supported by both participants A and B, who improved their performance on the post-test for the contrasting task. In addition, participants A and C improved on the Go/No-Go test. Interestingly, participant B showed large changes on TMT-A. For TMT-B, only participant A declined. While TMT-B is used to evaluate complex planning and working memory, TMT-A is employed to reflect simple planning ability, mental processing speed, and attention [37,44].

In terms of mean synchronization errors in the bimanual drum tapping tasks, the results were inconsistent among the three participants. The variability in these data may be due to the fact that age-related changes in sensorimotor processing for precise timing control can interfere with bimanual movements and can negatively impact one’s ability to match a changing tempo. Bimanual movements require greater neural resources for older adults [45], and high movement frequency during simultaneous (in-phase) and alternative (anti-phase) drum tapping may require an intense transition to playing with concurrent rhythmic cueing [46].

It is important to note that there were differences in how much time each participant spent engaging in the three types of drum playing (simultaneous, alternative, or mixed). This result implies that elderly participants in dual-task interventions need strategies to induce them to engage in the various tasks at equitable levels of engagement and support to monitor their participation. This study also highlights the need for future research to address the relationship between bimanual drumming engagement and EF. This is supported by the finding that participant B was less engaged in the more complex tasks of the intervention and showed the poorest performance on post-intervention EF measures. Considering the evidence on brain activity associated with cognitive–motor dual tasks [47], the limited attentional capacity in individuals with AD seems to interfere with intense and task-specific movements.

Another aspect of this dual-task-based music intervention is the intrinsic motivation involved in playing the drum. This has been documented in previous studies regarding motivation and participation in music activity [48]. Because mood is one of the predictors of progression to severe AD [49], it is important that patients with AD feel motivated and positive about their participation to maximize the benefits of their intervention [50].

In this study, we provided a dual-task-based music therapy intervention to octogenarians with early stage AD. It has been noted that clinical trials with patients with AD fail to include participants who represent the oldest old. However, the majority of patients with AD today are over 80 years old [51]. Since the majority of patients with AD are over 80 years of age, it is necessary to develop and test music therapy interventions that can effectively address their specific needs.

Drum playing, as a simple form of music making, is sufficiently complex to stimulate EF and can be considered an important therapeutic alternative for elderly patients with AD. For the elderly, physical movement can reduce the risk of dementia related to aging and may enhance cognitive activity by stimulating brain processes and protecting against neural decline [52]. Drum playing involves upper limb movements in the music-making process, and changing tempos and different types of playing activate cognitive flexibility. Previous studies show that one of the most beneficial aspects of music playing for individuals with AD is that music affects multiple domains of cognition [53,54,55]. It is meaningful to see that dual-task-based music therapy can be utilized to protect cognitive functioning in elderly patients with early stage AD. Our findings should be considered in light of some study limitations. Because we only included three cases, our conclusions must be tempered by s lack of generalizability.

## 5. Conclusions

This study evaluated the impact of a music therapy program based on the dual-task paradigm on improving the cognitive function of elderly patients with early stage AD. Drum tapping with live musical cueing served as the bimanual task. Although the post-test results indicate generally favorable outcomes, there are limitations associated with the results. Most notably, because only three participants were included in the study, the generalizability of the findings is limited. As such, this multiple case study should be expanded to include a larger sample size as well as a control group to confirm the statistical significance of the findings. In addition, this study included geriatric patients who have experienced deterioration associated with aging; therefore, future studies could employ standardized rhythm tests as an assessment tool to confirm the homogeneity of the participants’ characteristics.

Overall, we found that the EF of octogenarians with early stage AD could be enhanced by a dual-task-based music therapy intervention with varying levels of engagement. Further investigation is warranted to identify the various underlying mechanisms for how instrument playing can impact the performance of complex cognitive–motor tasks.

## Figures and Tables

**Table 1:** Participants’ demographic information.

Participant	Gender	Age (Years)	Education (Years)	Dominant Hand	K-CIST	SMCQ	GDS
A	Male	80	6	Right	12	5	8
B	Female	83	2	Right	8	12	17
C	Male	85	14	Right	14	4	1

Note. K-CIST = Korean version of the Cognitive Impairment Screening Test; SMCQ = Subjective Memory Complaints Questionnaire; GDS = Geriatric Depression Scale.

**Table 2:** The steps of the dual-task-based drum-playing intervention.

Step	Intervention Content	Time Required
1	▪ Introduction: Respiratory and muscle relaxation	5 min
2	▪ Drum playing based on the dual task ▪ Bimanual drum playing with regular rhythmic cueing: simultaneous, alternative, or mixed ▪ Phased drum-playing tasks with more complex rhythmic cueing and tempo changes	30 min
3	▪ Chanting with rhythmic cueing and therapeutic singing	5 min

**Table 3:** Changes in cognitive measurements.

Parameter	Pt.	Pre-Test	Post-Test	Change
Contrasting (count)	A	11	20	+9
	B	13	17	+4
	C	20	20	-
Go/No-Go (count)	A	8	18	+10
	B	8	6	−2
	C	9	11	+2
Fist–Edge–Palm (seconds)	A	(Rt) 27.53	(Rt) 23.72	(Rt) −3.81
		(Lt) 20.13	(Lt) 19.63	(Lt) −0.50
	B	Fail	Fail	-
	C	(Rt) 26.78	(Rt) 31.69	(Rt) +4.91
		(Lt) 22.65	(Lt) 27.35	(Lt) +4.70
Alternating hand movement (seconds)	A	11.22	8.81	−2.41
	B	Fail	Fail	-
	C	12.34	10.47	−1.87
TMT-A (seconds)	A	29.05	25.44	−3.61
	B	214.0	104.37	−109.63
	C	35.62	27.27	−8.35
TMT-B (seconds)	A	111.47	101.25	−10.22
	B	Fail	Fail	-
	C	207.57	257.00	+49.43

Note. Pt = participant; Rt = right; Lt = left; TMT = Trail Making Test.

**Table 4:** Mean synchronization errors for each tempo condition of the drum tapping task.

Pt.	Tempo Condition of Tapping Task									
	−20%		−10%		Self-Paced		+10%		+20%	
	Pre	Post	Pre	Post	Pre	Post	Pre	Post	Pre	Post
Simultaneous (M ± SD)										
A	0.032 ±0.018	0.025 ±0.019	0.021 ±0.018	0.028 ±0.023	0.013 ±0.012	0.040 ±0.025	0.020 ±0.011	0.034 ±0.036	0.029 ±0.018	0.017 ±0.009
B	0.063 ±0.031	0.036 ±0.033	0.030 ±0.024	0.050 ±0.034	0.021 ±0.026	0.063 ±0.050	0.059 ±0.023	0.053 ±0.014	0.052 ±0.044	0.016 ±0.025
C	0.026 ±0.021	0.086 ±0.028	0.058 ±0.046	0.029 ±0.020	0.041 ±0.041	0.013 ±0.010	0.044 ±0.024	0.011 ±0.007	0.024 ±0.024	0.021 ±0.018
Alternative (M ± SD)										
A	0.099 ±0.066	0.083 ±0.43	0.032 ±0.019	0.054 ±0.038	0.074 ±0.034	0.065 ±0.048	0.062 ±0.022	0.084 ±0.041	0.019 ±0.021	0.086 ±0.029
B	0.043 ±0.041	0.087 ±0.059	0.049 ±0.030	0.098 ±0.035	0.035 ±0.024	0.081 ±0.034	0.031 ±0.020	0.069 ±0.021	0.059 ±0.098	0.030 ±0.020
C	0.071 ±0.038	0.034 ±0.021	0.059 ±0.070	0.072 ±0.060	0.020 ±0.013	0.025 ±0.024	0.094 ±0.089	0.029 ±0.030	0.034 ±0.021	0.053 ±0.030
